# Effects of Low-Dose Drinking Water Arsenic on Mouse Fetal and Postnatal Growth and Development

**DOI:** 10.1371/journal.pone.0038249

**Published:** 2012-05-31

**Authors:** Courtney D. Kozul-Horvath, Fokko Zandbergen, Brian P. Jackson, Richard I. Enelow, Joshua W. Hamilton

**Affiliations:** 1 Department of Microbiology and Immunology, Dartmouth Medical School, Hanover, New Hampshire, United States of America; 2 Bay Paul Center for Comparative Molecular Biology and Evolution, Marine Biological Laboratory, Woods Hole, Massachusetts, United States of America; 3 Department of Earth Sciences, Dartmouth College, Hanover, New Hampshire, United States of America; 4 Department of Medicine, Dartmouth Medical School, Hanover, New Hampshire, United States of America; Sapienza University of Rome, Italy

## Abstract

**Background:**

Arsenic (As) exposure is a significant worldwide environmental health concern. Chronic exposure via contaminated drinking water has been associated with an increased incidence of a number of diseases, including reproductive and developmental effects. The goal of this study was to identify adverse outcomes in a mouse model of early life exposure to low-dose drinking water As (10 ppb, current U.S. EPA Maximum Contaminant Level).

**Methodology and Findings:**

C57B6/J pups were exposed to 10 ppb As, via the dam in her drinking water, either in utero and/or during the postnatal period. Birth outcomes, the growth of the F1 offspring, and health of the dams were assessed by a variety of measurements. Birth outcomes including litter weight, number of pups, and gestational length were unaffected. However, exposure during the in utero and postnatal period resulted in significant growth deficits in the offspring after birth, which was principally a result of decreased nutrients in the dam's breast milk. Cross-fostering of the pups reversed the growth deficit. Arsenic exposed dams displayed altered liver and breast milk triglyceride levels and serum profiles during pregnancy and lactation. The growth deficits in the F1 offspring resolved following separation from the dam and cessation of exposure in male mice, but did not resolve in female mice up to six weeks of age.

**Conclusions/Significance:**

Exposure to As at the current U.S. drinking water standard during critical windows of development induces a number of adverse health outcomes for both the dam and offspring. Such effects may contribute to the increased disease risks observed in human populations.

## Introduction

Chronic exposure to arsenic (As), by contamination of drinking water from natural geological sources, is a significant worldwide environmental health concern [1], [2]. As many as 25 million people in the United States are exposed to As at levels above the current EPA standard from private, unregulated wells, and worldwide the estimated exposure is several hundreds of millions of people. Chronic exposure to such elevated levels has been associated with a variety of adverse health impacts in human epidemiology studies, including various cancers, cardiovascular disease, diabetes and developmental/reproductive effects [2], [3], [4]. The mechanism(s) by which arsenic can induce or contribute to such a wide array of adverse health impacts, over a wide range of exposures, have yet to be elucidated.

Exposure events during critical windows of fetal and postnatal (PN) development pose a serious risk for adverse health outcomes later in life [5]. An increasing number of animal and human epidemiological studies have indicated an association between As exposure and adverse reproductive and developmental outcomes. Arsenic readily crosses the placenta [6], [7], but it has been shown that As transfer via the breast milk is limited [8]. Prenatal exposure to high levels of As (ppm range) in mouse models has been reported to result in a variety of adverse effects including placental dysplasia and loss of fecundity [9], transplacental carcinogenicity [10], early onset of atherosclerosis [11], and neural tube defects [12]. Prenatal exposure of mice to lower doses of As (50 ppb) has been shown to result in learning deficits [13] and alterations in lung structure and function [14].

Epidemiology studies have shown that exposure to As during gestation is associated with a number of adverse effects on the fetus, including low birth weight, survival, and spontaneous abortion [15], [16], [17], [18], [19]. However, most of this work has been conducted in areas with relatively high exposures (several hundred ppb), such as Bangladesh and Taiwan. A number of studies have identified adverse immunological outcomes following early life As exposure, including decreased infant thymic development, decreased immune factors in breast milk, increased acute respiratory infections in offspring, and the activation of inflammation in the placenta and cord blood [20], [21], [22], [23]. Early life As exposure has also been associated with an increased incidence of cancer and bronchiectasis in adulthood, even several decades after cessation of exposure [24]. Interestingly, in Bangladesh, no effects have been observed on infant development at 7 or 18 months [25], [26].

Based on cancer evidence, the U.S. EPA's current Maximum Contaminant Level (MCL) for As in public drinking water supplies is 10 ppb (0.13 µM) [27], which was recently revised from 50 ppb. Since that regulatory change, a number of studies have reported significant effects of As exposure at or below 10 ppb in experimental model systems [14], [28], [29], [30]. The effects of low dose As at the current EPA standard on susceptible populations, such as pregnant women and infants, have been largely unexplored. Our goal was to develop a mouse model of early life As exposure through which we could identify critical windows of exposure that might result in adverse impacts on the development of the immune system later in life. However, following the development of our model, we unexpectedly observed that gestational and post natal As exposure at 10 ppb caused immediate effects on the rate of body weight gain of the F1 offspring. Thus, the studies presented here were designed to further explore the developmental outcomes following low dose As exposure during gestation and lactation.

## Results

### Birth outcomes

**Figure 1:**
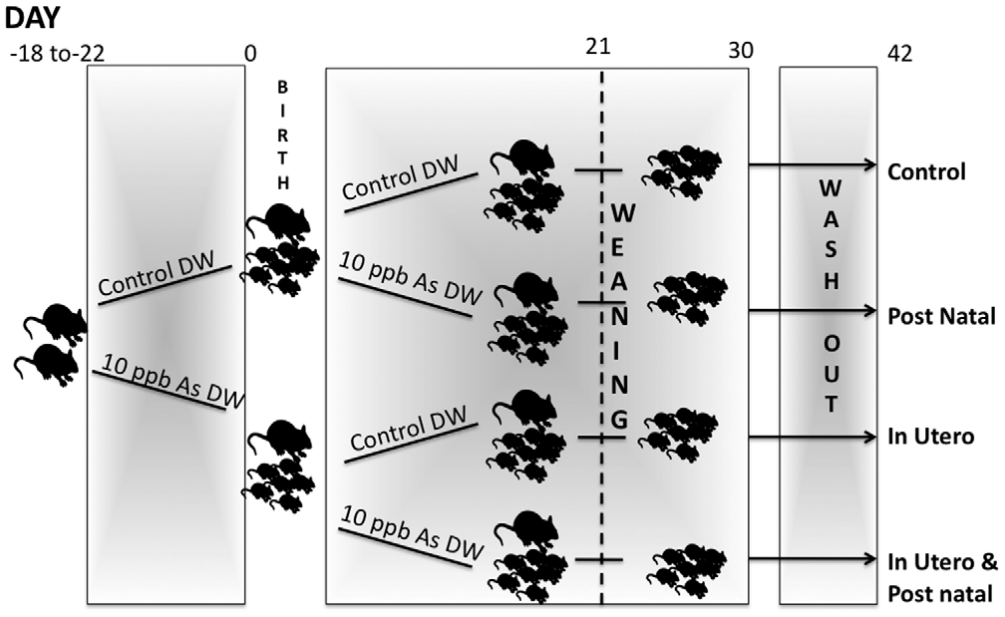
Experimental model of developmental arsenic exposure in C57BL/6 mice. Following the detection of cervical plugs, mated females were exposed to control water or 10 ppb As in drinking water through the gestational period. At the birth of their pups, dams in each exposure group were further divided into sub-groups receiving control water or 10 ppb As in drinking water through 30 days of age (n = 14–17 dams per exposure). Weaning from the dam took place at day 21 PN (or later if a pup did not reach the 7 gram weight cut-off). This resulted in 4 overall exposure groups: 1. Control (no As drinking water exposure; 2. Postnatal (PN, offspring receiving 10 ppb As from PN days 1–30); 3. In utero (IU, offspring receiving 10 ppb As from gestational day 1 through birth); 4. In utero & postnatal (IU&PN, offspring receiving 10 ppb As from gestational day 1 through day 30 PN) At day 30 PN, all offspring were placed on control drinking water and growth was assessed until 6 weeks PN.

The experimental model of exposure is detailed in Figure 1 and the Methods section. Differences were not observed in any birth outcome (n = 14–17 dams per exposure), such as litter size (control: 7.5 (±0.3) pups; arsenic: 7.4 (±0.3) pups), gestational length (control: 20.2 (±0.33) days; arsenic: 19.6 (±0.38) days), the average weight of the litter or the survival of the pups (control: 1.28 (±0.01) grams; arsenic: 1.26 (±0.02) grams).

### Growth of F1 offspring

**Figure 2:**
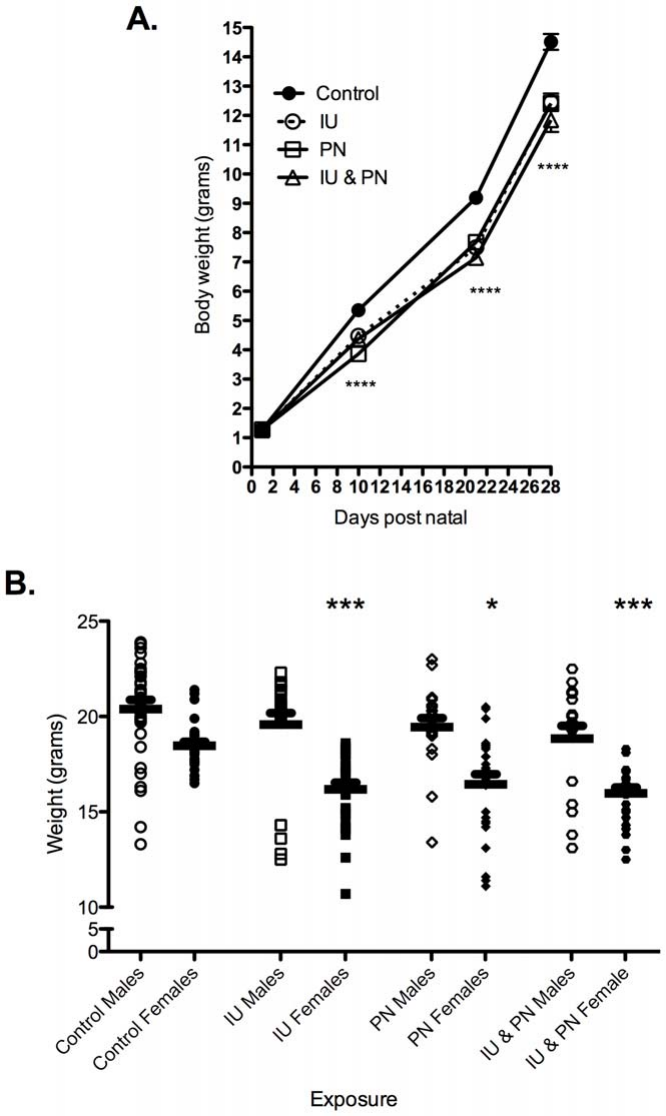
Effects of in utero and postnatal arsenic exposure on growth of offspring. (A). The weight (grams) of offspring was monitored over the course of development and is shown across all four exposure groups at birth, day 10, 21, and day 28 PN. Male and female mice are included. Birth, n = 21–25; Day 10, n = 17–21; Day 21, n = 65–71; Day 28, n = 44–52. One Way ANOVA, compared to control for each respective time point (B.) Mouse weights separated by gender at day 42 PN across all four exposure groups; n = 49–54. Male mice in all exposure groups are represented with open shapes and female mice with closed shapes. One Way ANOVA for female mice, compared to unexposed female mice. Asterisks indicate statistical significance, * p<0.05, ** p<0.01*** p<0.001, ****p<0.0001. Error bars represent mean ± SEM.

Following birth, pups were monitored daily to assess survival and development. Survival and developmental milestones (eye opening, pinna unfolding, appearance of fur) was not differentially affected by the As exposure paradigm. As early as day 10 post natal, offspring exposed to As displayed significant decreases in growth (evidenced by total body weight), regardless of the timing of As exposure (Figure 2A). At the time of weaning (day 21 post natal) many of the As-exposed offspring were so small that it was not feasible to separate the offspring from the dams at the day of weaning. All pups were maintained with the dam until they reached a weight standard for separation of 7 grams. At weaning, mean weight values for IU, PN and IU&PN As exposed offspring were 7.5, 7.6 and 7.1 grams, respectively. The mean control weight was 9.2 grams. Given this cutoff for separation from the dam, percentages of mice requiring a delayed wean for control, IU, PN and IU&PN exposures were as follows: 0%, 33%, 25% and 46%, respectively. This growth deficit persisted through day 28 PN, regardless of whether or not a delayed wean was required. At day 42 PN, the growth deficit was still apparent in female but not in male mice (Figure 2B). Prior to this time point, both genders of mice were equally affected (Figure S1).

### Arsenic tissue concentrations

To assess the direct exposure of the F1 offspring to As, total As levels in the placenta, dam's breast milk, pup stomach content (day 10 and 21 PN), and urine were measured by ICP-MS across all exposure groups at various time points pre-, during and post-pregnancy. Arsenic levels in the placentas, dam's milk and pup stomachs were near the limits of detection in all exposure groups. There was no observable increase in the As concentrations in the placenta or milk, but there was a significant increase in the stomach As contents of the pups that received As exposure IU and IU&PN compared to control offspring. This increase in stomach As levels was observed at day 10 PN, during which time the offspring would be primarily consuming breast milk, as opposed to drinking from the water bottle source of As. By day 21 PN, no differences in As levels were detected in the stomach contents of the pups. As expected, urinary excretion of As significantly increased in the As exposure groups. Interestingly, while all mice in the exposure groups received the same level of As (10 ppb), excretion values decreased in the pregnant and lactating As-exposed mice, when compared to As-exposed virgin mice. Mice that were exposed to As only IU no longer excreted significant levels of As during the postpartum period. Urinary As excretion was primarily in the form of dimethylarsenate (DMA). Values are represented in Table 1. Intake of water and excretion of urine displayed a trending, but not significant, increase across the exposure groups (Table S1).

**Table 1:** Arsenic measurements^a^ in tissue samples from dams and pups.

	Control	As (2 week)
**Virgin Mice**		
**Urine (ug/L)**	11.91 (5.0)	56.01 (13.1)***

	Control	IU
**Gestational Day 15.5**		
**Placenta (ng/g)**	1.20 (0.4)	1.00 (0.5)
**Dam's Urine (ug/L)**	6.89 (2.5)	26.75 (6.9)* ^##^

	Control	IU	PN	IU & PN
**PN day 10–12**				
**Pup stomach (ng/g)**	0.64 (0.3)	2.1 (0.5) **	1.17 (0.4)	2.69 (1.0) ***
**Dam's Milk (ng/g)**	1.56 (0.6)	0.89 (0.4)	0.78 (0.5)	1.73 (1.0)
**Dam's Urine (ug/L)**	4.41 (2.5)	5.94 (3.3)	19.68 (3.1)*** ^###^	20.00 (1.3)*** ^###^
**PN day 21**				
**Pup stomach (ng/g)**	4.8 (0.4)	5.0 (0.3)	4.6 (0.9)	3.7 (0.8)

a Total As levels were measured by ICP-MS, as described in Methods (n = 3–6). Values represent mean ± SEM. Asterisks indicate statistical significance from respective control group in matching row.

* p<0.05,

** p<0.01,

*** p<0.001. Number sign indicates statistical significance from virgin female mouse exposed to 10 ppb As for 2 weeks. One Way ANOVA.

### Alterations in breast milk nutrition

**Figure 3:**
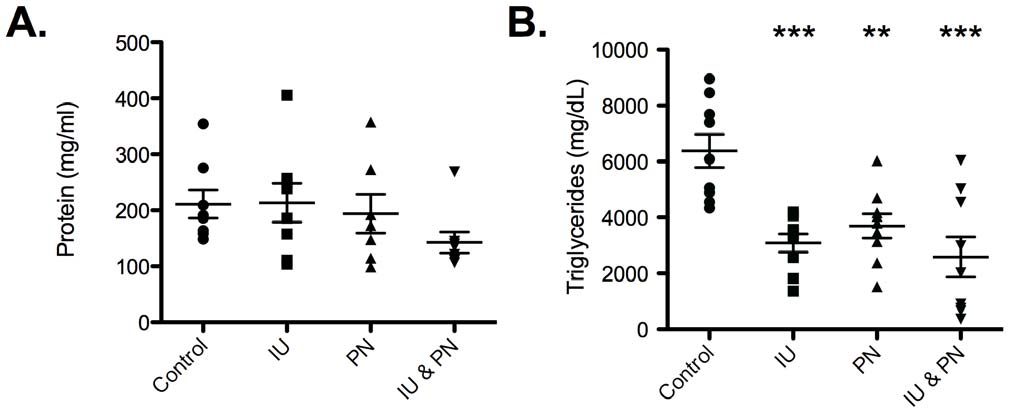
Effects of arsenic exposure on nutritional composition of dam's breast milk. Breast milk was collected between day 10–12 PN. Breast milk was analyzed for (A.) total protein concentrations and (B.) total TG concentrations. Asterisks indicate statistical significance, ** p<0.01, *** p<0.001, One Way ANOVA compared to control. Error bars equal mean ± SEM (n = 8–9 mice per exposure).

Given that the growth deficit manifested during the postnatal period (as early as day 10) and there was not a significant increase in the exposure of the pups to As via the breast milk, we hypothesized that other alterations in the dam's milk were at least partially responsible. Obvious differences in the rearing of the pups by the dams in different exposure groups were not observed. By day 1 PN, all pups displayed prominent milk spots and by day 10 PN, no significant difference was observed in the weight of the stomach contents of the pups, which confirmed that the offspring in all exposure groups were being fed and general milk production was intact. The milk of the dams in all exposure groups was assessed for total protein and triglyceride (TG) concentrations. Protein concentrations were not significantly different (Figure 3A), but TG concentrations displayed a significant decrease in all As exposure groups compared to control (Figure 3B). No differences were observed in the amount of milk collected in the different exposure groups.

### Triglyceride levels in the dams

**Figure 4:**
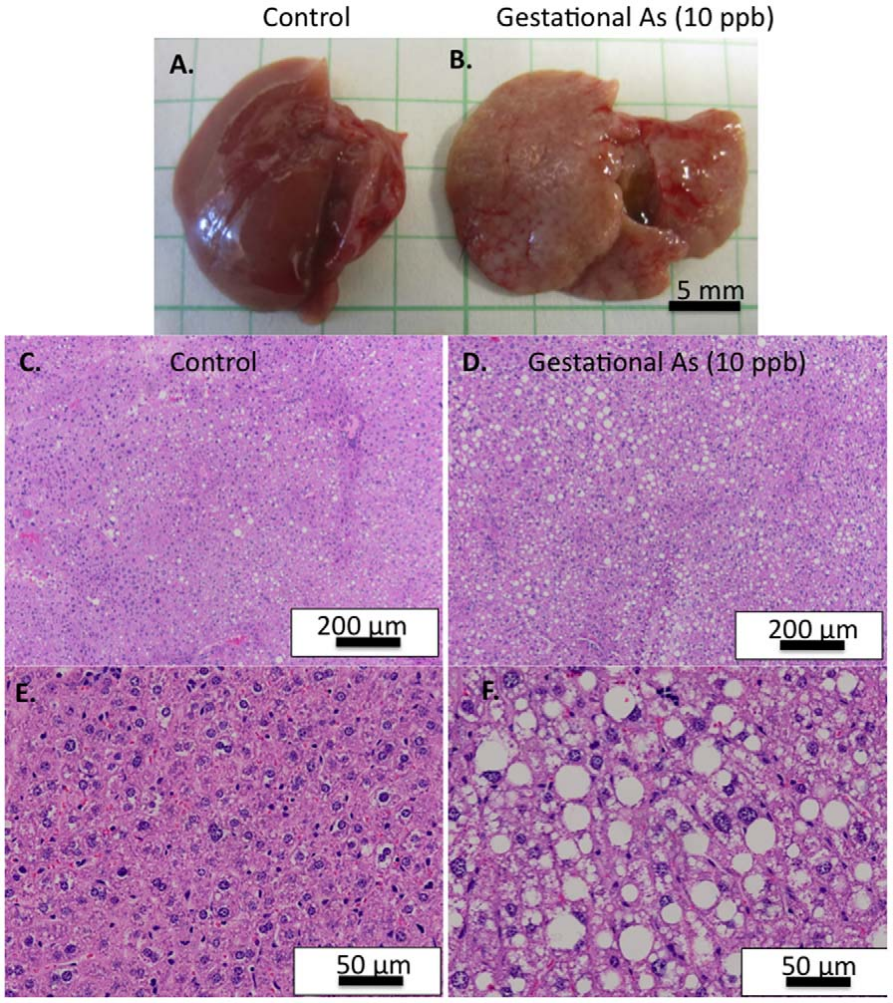
Effects of gestational arsenic exposure on liver steatosis and gross organ changes in the dam. Dams were sacrificed at gestation day 15.5. (A, B.) Detection of liver steatosis at the gross level was observed in a significant percentage of the As exposed mice (C–F.) Histological hematoxylin and eosin staining in (C.,E.) control and (D., F.) As-exposed dams. Scale bars indicate magnification.

We were interested to assess how exposure to As might alter lipid metabolism in the dams during the course of pregnancy and lactation. Unexpectedly, we found a significant increase in the incidence of liver steatosis in the As-exposed dams at day 15.5 gestation. None of the control dams displayed gross liver steatosis at day 15.5 gestation, while 55.5% (5/9) of the As exposure dams displayed gross liver steatosis (* p<0.05, compared to control, Fisher's exact probability test). Liver steatosis was confirmed at the histological level (Figure 4 A–F). Maternal weight gain was not altered by As exposure (Figure S2). Gross liver steatosis was not observed at day 21 PN in any of the exposure groups, indicating that the effects are transient. The total TG levels in the serum and livers of pregnant (gestational day 15.5) and lactating mice (PN day 15–18) were also measured (Table 2). Significant decreases were observed in the levels of total serum TG during pregnancy and lactation for mice receiving As in all exposure paradigms. Liver TG were significantly increased in mice exposed to As during gestation, as expected, given the gross liver steatosis. However, liver TG levels displayed a trending, but not significant, increase in As exposure groups during the lactation period.

**Table 2:** Exposure to Arsenic during gestation and lactation affect total TG levels in the serum and liver.

	Control	IU
**Gestational Day 15.5**		
**Serum TG (mg/dL)**	119 (8.5)	74 (0.5)**
**Liver TG (mg/dL/mg liver)**	4.1 (0.1)	6.85 (0.7)*

	Control	IU	PN	IU & PN
**PN day 15–18**				
**Serum TG (mg/dL)**	110 (10.3)	70 (4.0)*	71 (9.9)*	65 (5.7)*
**Liver TG (mg/dL/mg liver)**	4.2 (0.6)	6.1 (1.4)	5.3 (0.7)	5.44 (0.4)

Asterisks indicate statistical significance,

* p<0.05 and

** p<0.01, compared to respective control. Values represent mean ± SEM. Two tailed student's t-test for gestational exposure; One Way ANOVA for post natal exposures.

**Figure 5:**
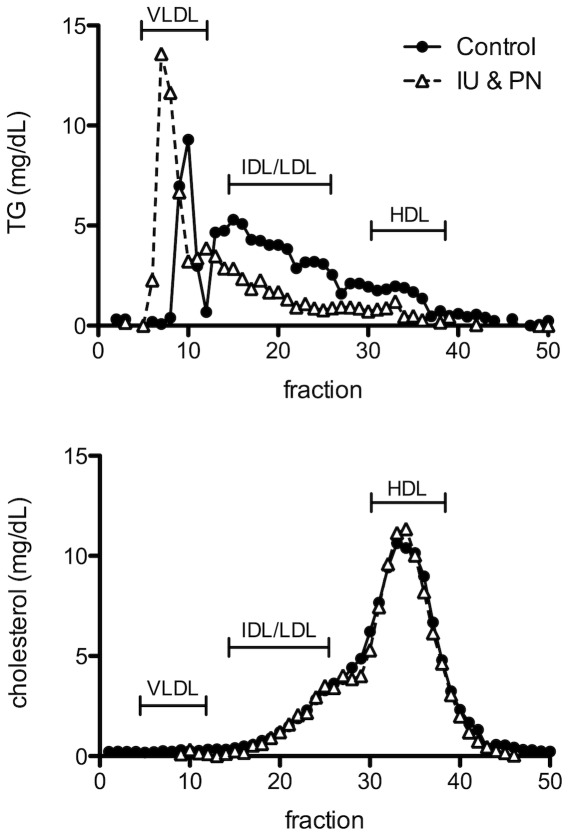
Effects of arsenic exposure on the distribution of cholesterol and triglycerides over serum lipoprotein fractions. Dams were fasted for 6 hours on PN day 21 and pooled serum of 4 As (IU&PN) treated dams (open triangles) and 3 controls (black circles) was used for lipoprotein profiling by FPLC. Fractions were assayed for TG (top panel) and total cholesterol levels (bottom panel).

Profiling of serum lipoproteins using Fast Protein Liquid Chromatography revealed no clear differences between the control and As-exposed dams for cholesterol levels in the lipoprotein fractions, indicating that 10 ppb As does not substantially affect the number of lipoprotein particles for any of the fractions (Figure 5). Interestingly, the distribution of TG over the fractions was markedly affected. The very low density lipoprotein (VLDL) fractions of the As-treated mice contained more TG and its distribution was shifted to larger VLDL particles. The low, intermediate, and high density lipoprotein fractions (LDL, IDL, and HDL, respectively), all showed a decreased TG level for the As-treated dams. In accordance with the results from Table 2, the area under the curve for TG was smaller for the As-treated mice (Figure 5).

### Cross-fostering of F1 offspring

**Figure 6:**
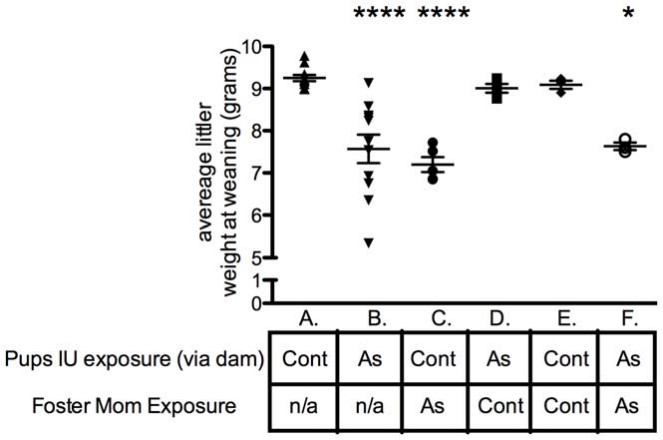
Effects of cross-fostering on postnatal growth of in utero arsenic-exposed pups. The average litter weight (grams) of offspring at weaning (day 21 PN) was assessed following fostering. Immediately following birth, all dams were placed on control drinking water through the weaning period. Groups were as follows: (A.) Control offspring remaining with biological mom through weaning/not fostered (n = 11 litters); (B.) 10 ppb IU As exposed mice remaining with biological mom/not fostered (n = 11 litters); (C.) Control offspring fostered by 10 ppb IU As mom (n = 5 litters); (D.) 10 ppb IU As offspring fostered by control mom (n = 5 litters); (E.) Control offspring fostered by a non-biological control mom (n = 3 litters); (F.) 10 ppb IU As offspring fostered by a non-biological 10 ppb IU As mom (n = 3 litters). Asterisks indicate statistical significance, * p<0.05, **** p<0.0001, One Way ANOVA compared to control mice remaining with biological mom (column A). Error bars represent mean ± SEM.

To assess the level to which decreased milk nutrients contributed to the growth deficit, we designed a series of cross-fostering experiments. In these experiments, offspring from opposing gestational exposure groups (control or gestational As) were exchanged and fostered. We observed that we could reverse the growth phenotype by simply exchanging the litters and dams from opposing exposure groups (Figure 6, Column C and D). Fostering within the exposure group recapitulated the original phenotype (Figure 6, Column E and F).

## Discussion

In the conduct of an experiment designed to examine immune effects later in life from in utero exposure to As at the current U.S. EPA drinking water standard, we were surprised to observe significant short-term effects of these exposures on the F1 offspring and the dams. Specific effects included decreased growth of the F1 offspring and altered TG levels and profiles in the dams. Decreases in the nutrient content of the dam's breast milk, specifically TGs, appear to play a role in the growth deficits observed in the F1 offspring. The growth deficit was reversed by cross-fostering of the pups.

Arsenic concentrations were measured in the dam's milk, pup stomachs, placentas, and dam's urine, which confirmed that As was not transferred via the breast milk. In two exposure groups (IU and PN), we observed a trend towards decreased As levels in the milk, which was also recently reported in a similar mouse study with much higher doses of exposure [31]. These results are consistent with published reports of healthy lactating women exposed to As [8]. We did not detect significant increases in the levels of As in the placentas of the exposed mice compared to control mice. It is possible that As transfers through the placenta at a greater level in the exposed mice, but the low level of As used in this model and the detection limits of the ICP-MS impair our ability to measure it. Arsenic has been shown to transfer through the placenta in human models of exposure and in mouse models with higher levels of exposure [6], [7], [32]. Given the dramatic effect on growth in the offspring, we were concerned that the pups may be acquiring exposure to As from another external source, such as the dam's urine. To assess such exposure, we also measured the stomach content of the pups. While we did not see a significant increase in the As levels in the milk, we did see a significant increase in the As levels the pup stomachs in two exposure groups: IU and IU&PN at day 10 PN. However, it is unlikely that these elevated levels are a result of external exposure, via the urine or some other source, given that we did not observe such increases in the PN group. The dams in the PN exposure group excreted a significantly higher level of As in the urine compared to the IU group during the postnatal period. Thus, the basis for this increase in stomach As in these two groups is unclear. The overall levels of As in the stomachs at day 21 were increased compared to day 10 PN. This was likely a result of the pup's consumption of chow at this time point.

Interestingly, we also observed significant decreases in As excretion by the dams in all As exposure groups during the gestational and postnatal period, compared to virgin mice ingesting the same level of As. There was a trend towards increased water consumption and urine output in the As-exposed mice during pregnancy (and across all groups during the lactational period), but given the magnitude of these trends, the changes in water consumption and urine output are unlikely to account entirely for the significant decrease in urinary As output. Recent epidemiological data has shown that pregnancy can significantly alter the metabolism of As [33], but one challenge in the field has been extrapolating animal model studies of As exposure to human studies because mice are very efficient at metabolizing As [34]. Further research in this area is needed, but these data suggest that urinary As levels in pregnant mice may not be an accurate marker of the level of drinking water exposure.

The impeded growth of As-exposed offspring observed in this study was unexpected, given the low dose (10 ppb) of As used. Similar growth deficits have been observed in a high dose [10, 50 and 100 mg/L] IU exposure model of rats [35]. Other studies have used similar models of exposure (ppb and ppm levels of As during gestation and lactation), but have not reported changes in body weight gain early in life [10], [14], [32]. It has become clear in comparing different experiments that the dose of As is very important, as well as understanding and controlling the background concentrations of As in the diet and bedding [36]. Moreover, arsenic has been shown to display complicated, multi-phasic dose response curves over a broad range that suggest different physiological effects at different dose ranges [37], so in retrospect it is not surprising to observe a different effect at these lower exposures, which might then be superseded by other effects at higher doses. We also recognize that many of these effects appear to be transient and can be experimentally ameliorated, suggesting that differences in the timing of exposure and in the experiment endpoints between this study and other studies using similar models could influence the observed results. It is also interesting that we did not observe greater effects in the groups with the combined exposure period (i.e., IU+PN) versus groups with only one or the other period of exposure (IU or PN). We cannot rule out the possibility that there is some capability to adapt to such low level exposures. Clearly, the response curves of As exposure, particularly at low levels, are complex, but these unexpected low-level effects on fetal growth warrant further investigation.

Based on our results, it is clear that As effects on the dam play a major role in the growth deficit of the offspring. The results of the fostering experiments suggest that the milk is the major contributor to the effects we have observed. However, we cannot definitively rule out that small behavioral changes in the dams rearing of the pups did not play a role in the growth deficit. Breastfeeding of infants in human populations with As exposure has been shown to be protective against increased As exposure to the infant [38]. Our results suggest that As exposure can impact the composition and quality of breast milk, even when As is not being directly transferred via the breastmilk. We also observed an interesting gender-specific effect. These results suggest that female mice are more vulnerable to early life As exposure, compared to their male siblings, implying a hormonal contribution. Arsenic is a well-documented endocrine disruptor and this could represent yet another example of such actions in vivo [37], [39], [40], [41].

We have previously reported that a combined exposure of adult mice to As followed by a sublethal infection with influenza significantly increased the severity of respiratory infection [42]. Based on our previous studies, we have hypothesized that exposure to low levels of As may act as a predisposing factor, in which a secondary stress is needed to induce the adverse health effects we have observed. In the results reported in this study, we believe that pregnancy/lactation can act as such a secondary stress in the dams. Under conditions of physiological stress, such as during pregnancy and lactation, small alterations in lipid metabolism may be exacerbated, leading to the measureable decreases in the TG concentration of the breast milk, altered lipid profliles and the liver steatosis we observed in this study. The shift to increased VLDL size in As-treated dams, as indicated by FPLC analysis, may reflect an increase in hepatic *de novo* lipogenesis [43], which would be in agreement with the fatty livers observed in a significant number of the As-exposed dams. Alternatively, As exposure may result in impaired VLDL-TG catabolism, but this would be expected to result in elevated serum TG levels. It is conceivable that As affects serum lipid metabolism via multiple mechanisms with lower TG levels as the ultimate effect. An elevated serum TG by increased production or decreased catabolism of VLDL-TG may for instance be compensated by increased hepatic clearance of IDL/LDL triglycerides, which would be supported by the lower TG levels in the corresponding fractions from the As-exposed dams. Regardless, the lower serum TG levels would potentially reduce availability of lipids in peripheral tissues, including mammary glands. Acute fatty liver of pregnancy (AFLP) is a specific clinical disease reported in a small percentage of human pregnancies [44]. It is interesting to speculate that such a background of altered lipid metabolism, itself elaborated by the physiological stress of pregnancy, may be further altered by As.

Overall, this study indicates that low-level As exposure during the IU or PN period results in a number of immediate adverse effects, including altered TG levels and profiles and growth deficits in the F1 offspring. Many of these outcomes were direct effects on the dam, which manifested as adverse outcomes in the F1 offspring. The results suggest that exposure of vulnerable populations to As, perhaps at levels as low as the current MCL of 10 ppb, may induce a significant increase in adverse health outcomes, which has been previously unrecognized. It is well documented that exposure to chemicals and environmental toxicants during the developmental period can have both immediate and long-term health problems. The goal of our future research will be to address the impact of early-life As exposure on the development and relative adverse health risks of these F1 individuals as adults, and on their F2 offspring.

## Methods

### Animal husbandry

All animal studies were conducted in accordance with Association for Assessment and Accreditation of Laboratory Animal Care (AALAC)-approved guidelines using a protocol approved by the Dartmouth Institutional Animal Care and Use Committee (IACUC). Approval protocol number for Dartmouth IACUC was 10-10-02. All animals were treated humanely and with regard for alleviation of suffering. C57BL/6J mice (Jackson Labs, Bar Harbor, ME) were housed in ventilated cages with AIN-76A chow (Harlan Teklad, Madison, WI, ad lib) and corncob and carefresh bedding (Scott's Distributing, Hudson, NH). Background As concentrations in the diet were less than 20 ppb, which is a level too low to speciate. We have demonstrated in previous studies that the bioavailable fraction of inorganic arsenic in this diet is low and that we can clearly distinguish an experimental signal as compared to control by exposing animals to drinking water As at 10 ppb [36].

Mated mice were acclimated on the AIN-76A diet for 2 weeks prior to mating. In this model, following the detection of cervical plugs, naïve pregnant mice were exposed to control or 10 ppb As in drinking water through the gestational and weaning period. Males were not exposed to As prior to mating. At birth, dams in control and As exposure groups were further split into groups receiving control or 10 ppb As in drinking water, which resulted in 4 overall exposure groups: 1. Control (no As drinking water exposure); 2. Postnatal (PN, offspring receiving 10 ppb As from PN days 1–30); 3. In utero (IU, offspring receiving 10 ppb As from gestational day 1 through birth); 4. In utero & postnatal (IU&PN, offspring receiving 10 ppb As from gestational day 1 through day 30 PN). (Figure 1). Starting at day 18 gestation, cages were monitored twice daily for the birth of pups. Litters were not culled. Litters were monitored for developmental milestones, such as eye opening, pinna unfolding and the appearance of fur. Offspring were weaned from the mothers at day 21 PN and continued with their designated drinking water exposure through day 30 PN. From days 30–42 PN, all offspring were maintained on deionized distilled water ddH_2_O (no As). In all instances (dams and offspring), mice not being exposed to As during specified time frames, were given ddH_2_O. Background As levels in the ddH20 were below 1 ppb. Animals were euthanized with carbon dioxide. Maternal necropsies were conducted at day 15.5 gestation or at various time points during lactation. F1 offspring necropsies were conducted at day 10, 21 and 42 PN. No gross lesions or visceral internal malformations (observed by gross physical observation at necropsy) were noted in the FI offspring at any time point up to the termination of the study at day 42 postnatal. A subset of dams and virgin mice from each exposure group (n = 3–6) was placed in metabolic cages (Nalgene) for a period of 24 hours at indicated time points to facilitate collection of urine and measurement of drinking water intake (Table S1).

### Cross-fostering of pups

Litters born to a control dam or a gestationally As-exposed dam within 12 hours of each other were eligible for cross-fostering. Fostering of litters always occurred within 12 hours of birth. All biological offspring were removed, the litters were culled to seven pups, and they were transferred, as a group, to the cage of the foster dam. The behavior of the dam was monitored for one hour post-fostering to ensure acceptance of the litter. All dams were maintained on deionized distilled water ddH_2_O (no As) following birth. Weight (grams) of fostered offspring was assessed at day 21 PN.

### Arsenic exposure

Sodium arsenite (Sigma Aldrich, St. Louis, MO) was prepared from stock solution in ddH_2_O to yield a 10 ppb (µg/L) concentration of drinking water. The arsenic concentration in the final solution was confirmed by induction-coupled plasma mass spectrometry (ICP-MS) metal analysis at Dartmouth's Trace Elements Analysis Core Facility. Drinking water was changed twice weekly.

### Offspring growth

At birth, the gestational length, number of pups, average litter weight (weight of individual pups/number of pups in litter), number of dead pups, and number of pups with malformations were recorded. Weight (grams) was recorded for offspring at birth, day 10, day 21, day 28 and day 42 PN.

### Liver histology

Liver steatosis in the dams was observed at the gross observational level. For histological confirmation, livers were removed and fixed in formaldehyde, paraffin embedded, sliced and stained with hematoxylin and eosin.

### Collection of breast milk

Dams (day 10–12 postnatal) were separated from pups for 6 hours to allow for milk accumulation. Dams were lightly anesthetized (i.p.) with 9∶1 ketamine∶xylazine mix at 0.1 ml/30 g body weight and injected (i.p.) with 2 IU (100 uL) of oxytocin (Sigma-Aldrich, St. Louis, MO). Dams were milked by gentle manual stimulation of the teat and collection with a pipette. Milk was stored at −20 degrees C.

### Analysis of protein levels

Milk samples were diluted in PBS and assayed for protein (1∶400 dilution). Protein concentrations were determined by BCA Protein Assay (Pierce, Rockford, IL), according to manufacturer's instructions.

### Collection of serum and livers

Dams were fasted for 6 hours. Blood was collected from the vena cava and livers were collected and snap frozen. Liver tissue (∼150 mg) was homogenized in PBS using the Bullet Blender Homogenizer (Next Advance, Cambridge, MA).

### Analysis of triglycerides

Triglyceride concentrations were determined in the dam's breast milk (1∶160 dilution), liver tissue (1∶20 dilution) and serum (1∶2 dilution) samples from pregnant and lactating dams across all exposure groups, using Wako L-type triglyceride (Wako Diagnostics, Richmond, VA), according to manufacturer's instructions.

### Lipoprotein profiling

Lipoproteins were separated using fast protein liquid chromatography (FPLC). 0.2 mL of pooled mouse plasma was injected onto a Superose 6B 10/30 column (GE Healthcare Life Sciences, Piscataway, NJ) and eluted at a constant flow of 0.2 mL/minute with phosphate buffered saline (pH 7.4). The effluent was collected in 0.2 mL fractions and triglyceride and cholesterol levels were determined for each fraction using L-type and Cholesterol E kits from Wako Diagnostics (Richmond, VA).

### ICP-MS metal analysis

Total concentrations of trace metals in the breast milk (n = 4–5), pup stomachs (n = 6), maternal urine (n = 3) and placentas (n = 3) were measured by ICP-MS. Placenta, stomach and milk samples were digested with 0.3–0.5 ml optima HNO_3_ at 70°C followed by addition of 0.05–0.1 ml of H_2_O_2_. The digested samples were then diluted to 3 ml (stomach and milk) or 5 ml (placenta) final volume. These samples were then analyzed by ICP-MS (7700x, Agilent, Santa Clara, CA) using He as a collision gas for As determination. Quality control included sample analysis duplicates and spikes. Urine was diluted 10-fold with 1% optima HNO_3_ and analyzed for As by ICP-MS as above.

### Statistical analysis

Statistical analysis was performed with Graphpad Prism 5.0d for Macintosh (GraphPad Software Inc., La Jolla, CA) using two tailed student t-test, ANOVA (with Bonferroni post test) or Fisher's exact probability test, requiring p<0.05 for statistical significance.

## Supporting Information

Figure S1 **Effects of in utero and postnatal arsenic exposure on the gender specific growth of offspring.** Mouse weights separated by gender at (A.) day 21 and (B.) day 28 across all four exposure groups. No significant differences are observed when comparing male vs female mice within the same exposure group. n = 24–32.
(TIFF)

Figure S2 **Gestational As exposure does not affect weight gain in dams.** Maternal weight gain (n = 9) was not affected by As exposure (solid lines). The growth of virgin female mice was also not affected by As exposure (dotted lines). Error bars represent mean ± SEM.
(TIFF)

Table S1 **Water consumption and urine output in virgin, pregnant and lactating mice.**
(DOCX)
