# Supplementary figures and images for: Effects of Low-Dose Drinking Water Arsenic on Mouse Fetal and Postnatal Growth and Development
Source: PLoS One. 2012 May 31;7(5):e38249. doi: 10.1371/journal.pone.0038249 (PMC3365045; doi:10.1371/journal.pone.0038249)

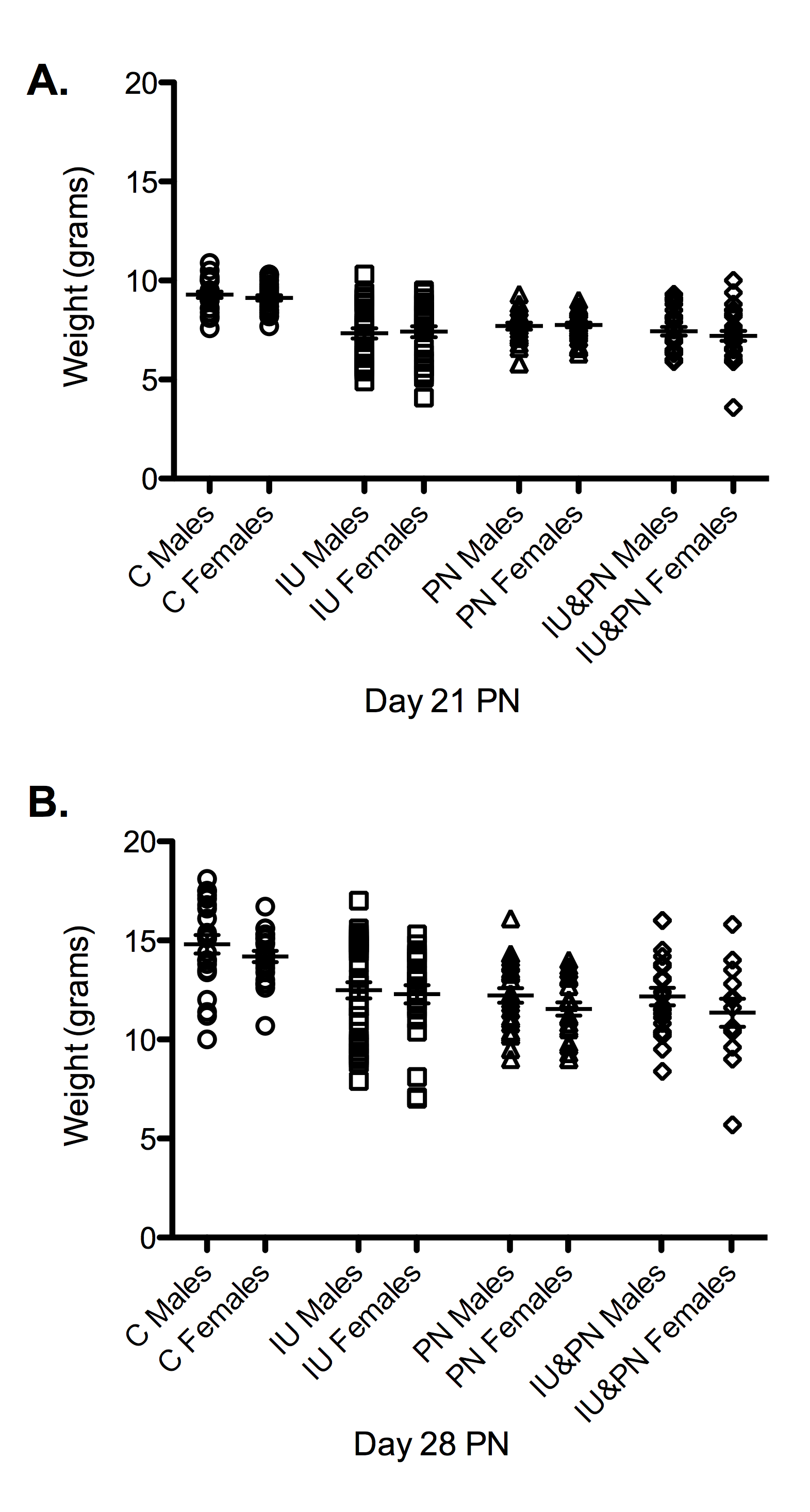

Supplement: Figure S1 — Effects of in utero and postnatal arsenic exposure on the gender specific growth of offspring. Mouse weights separated by gender at (A.) day 21 and (B.) day 28 across all four exposure groups. No significant differences are observed when comparing male vs female mice within the same exposure group. n = 24–32. (TIFF) [file pone.0038249.s001.tif]

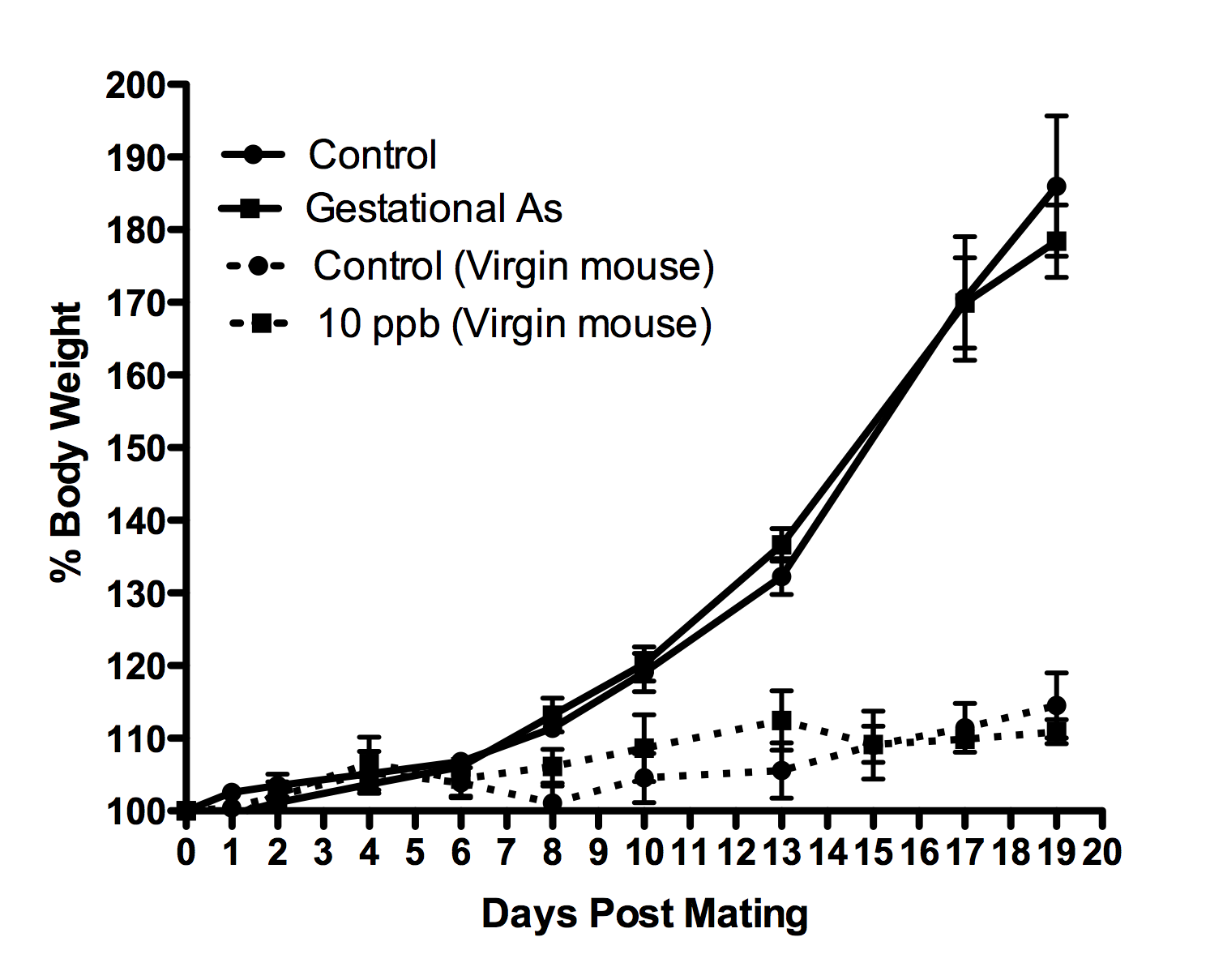

Supplement: Figure S2 — Gestational As exposure does not affect weight gain in dams. Maternal weight gain (n = 9) was not affected by As exposure (solid lines). The growth of virgin female mice was also not affected by As exposure (dotted lines). Error bars represent mean ± SEM. (TIFF) [file pone.0038249.s002.tif]
