# Supplementary material for: Effects of Low-Dose Drinking Water Arsenic on Mouse Fetal and Postnatal Growth and Development
Source: PLoS One. 2012 May 31;7(5):e38249. doi: 10.1371/journal.pone.0038249 (PMC3365045; doi:10.1371/journal.pone.0038249)
Supplement: Table S1 — Water consumption and urine output in virgin, pregnant and lactating mice. (DOCX) [file pone.0038249.s003.docx]

|  | **Control** | **As (2 week)** |  |  |
| --- | --- | --- | --- | --- |
| **Virgin Mice** |  |  |  |  |
| **Water consumption (mL)** | 4.2 (0.2) | 3.9 (0.6) |  |  |
| **Urine volume (mL)** | 1.0 (0.03) | 1.0 (0.02) |  |  |
|  | **Control** | **IU** |  |  |
| **Gestational Day 15.5** |  |  |  |  |
| **Water consumption (mL)** | 4.1 (0.6) | 5.4 (0.4) |  |  |
| **Urine volume (mL)** | 1.0 (0.05) | 1.4 (0.4) |  |  |
|  | **Control** | **IU** | **PN** | **IU & PN** |
| **PN day 10-12** |  |  |  |  |
| **Water consumption (mL)** | 4.8 (0.5) | 4.0 (0.4) | 4.5 (0.7) | 4.2 (0.9) |
| **Urine volume (mL)** | 2.5 (0.6) | 1.8 (0.7) | 1.8 (0.7) | 1.7 (0.4) |

Supplementary Table 1. Water consumption and urine output in virgin, pregnant and lactating mice.

Values represent mean ± SEM (n=3-5 mice per exposure). No significant differences were found by One Way ANOVA for As exposed groups compared to relative control.
